# Physician-Patient Communication about Novel Drugs and High-Risk Medical Devices

**DOI:** 10.1177/0272989X241302096

**Published:** 2024-12-21

**Authors:** Sanket S. Dhruva, Aaron S. Kesselheim, Steven Woloshin, Robin Z. Ji, Zhigang Lu, Jonathan J. Darrow, Rita F. Redberg

**Affiliations:** University of California, San Francisco School of Medicine, San Francisco, CA, USA; Philip R. Lee Institute for Health Policy Studies, University of California, San Francisco, CA, USA; Section of Cardiology, Department of Medicine, San Francisco Veterans Affairs Medical Center, San Francisco, CA, USA; Program On Regulation, Therapeutics, And Law (PORTAL), Division of Pharmacoepidemiology and Pharmacoeconomics, Department of Medicine, Brigham and Women’s Hospital and Harvard Medical School, Boston, MA, USA; Dartmouth Institute for Health Policy and Clinical Practice, Lebanon, NH, USA; University of California, San Francisco School of Medicine, San Francisco, CA, USA; Division of Cardiology, Department of Medicine, University of California, San Francisco School of Medicine, San Francisco, CA, USA; Program On Regulation, Therapeutics, And Law (PORTAL), Division of Pharmacoepidemiology and Pharmacoeconomics, Department of Medicine, Brigham and Women’s Hospital and Harvard Medical School, Boston, MA, USA; Program On Regulation, Therapeutics, And Law (PORTAL), Division of Pharmacoepidemiology and Pharmacoeconomics, Department of Medicine, Brigham and Women’s Hospital and Harvard Medical School, Boston, MA, USA; University of California, San Francisco School of Medicine, San Francisco, CA, USA; Philip R. Lee Institute for Health Policy Studies, University of California, San Francisco, CA, USA; Division of Cardiology, Department of Medicine, University of California, San Francisco School of Medicine, San Francisco, CA, USA

**Keywords:** drugs, medical devices, food & drug administration, physician-patient communication, adverse events

## Abstract

**Background:**

After a new drug or medical device is approved by the US Food and Drug Administration (FDA), physician-patient communication about benefits and risks is critical, including whether the product was approved through an expedited pathway based on limited evidence. In addition, physician reporting of drug- and device-related adverse events in real-world use is necessary to have a complete safety profile. We studied physician-reported communication and safety-reporting practices related to drugs and devices.

**Methods:**

We surveyed a random national sample of American Board of Internal Medicine–certified internists, cardiologists, and oncologists between October 2021 and September 2022 about the sources of information used to prescribe a drug or medical device, details of communication with patients, and reporting of adverse events.

**Results:**

Among 509 respondents (39% response rate), 387 (76%) reported that FDA approval influenced their decision “a lot” to prescribe a new drug or recommend use of a medical device. Half (122; 50%) of the 244 physicians randomized to receive a question about their own communication of trial endpoints reported “usually” telling patients when products were approved based on surrogate measures and 126 (52%) “usually” reported telling patients if a postapproval trial was required to evaluate safety and effectiveness. Two-thirds (165) said they were likely to report drug- or device-related adverse events to FDA.

**Conclusions:**

Physician self-reporting of communication with patients about drugs and devices suggests that half include characteristics of the pivotal trials such as use of clinically meaningful endpoints or continued requirement for evidence generation.

**Implications:**

More consistent discussions with patients about the quality of evidence supporting new drugs and devices and increased reporting of adverse events could ensure optimal use of these products in clinical practice.

**Highlights:**

- Among 509 board-certified internists, cardiologists, and oncologists, half reported telling patients when drugs or medical devices were approved based on surrogate measures and when there was an FDA-mandated postapproval trial to further evaluate safety and effectiveness.
- As drugs and medical devices are increasingly approved by the FDA through expedited pathways based on data with lingering uncertainties, discussion with patients about issues such as the nature of the endpoints assessed and existence of postapproval testing requirements can help inform patient decision making.

New drugs and high-risk medical devices (e.g., transcatheter heart valves, deep brain stimulators) offer therapeutic options for patients but are associated with risks. Physician-patient communication about the evidence for these products is crucial for informed and appropriate decision making, a necessary underpinning of patient autonomy.^1^

While patients may have variability in their desire for information, ensuring that physicians communicate benefits and risks is particularly important when insufficient evidence is available. With increasing use of expedited pathways for new drugs and devices, the US Food and Drug Administration (FDA) has been trading faster approval for more limited clinical evidence about a drug’s or device’s safety and efficacy.^2–5^ For example, for a growing number of drugs and devices, these expedited pathways lead to marketing based on trials showing changes in surrogate measures,^2^ which are markers that are expected to predict clinical outcomes.^6^ The FDA suggests remaining uncertainty and key questions can be addressed through postapproval studies, as part of a “total product life-cycle” approach.^7–9^

When drugs and medical devices are used in the postmarket setting in larger numbers of patients and with longer follow-up than in the more limited trials supporting expedited FDA authorization, more adverse events will inevitably be discovered with better precision about adverse event rates. Drugs and devices approved through expedited pathways are more likely to have new postapproval boxed warnings or contraindications added to their labeling than those going through the traditional approval process.^10–13^ Postmarket surveillance is critical to expanding public knowledge about medical product safety and patient decision-making autonomy but can be limited by delays in the completion of postmarket trials^8,14–20^ and underreporting of adverse events.^21^ As few as 3% of adverse events may be reported to the FDA.^22^

To better understand how these factors contribute to physician-reported communication about new drugs and devices, we surveyed a national sample of internists, cardiologists, and oncologists about what evidence sources inform their decisions to recommend drugs or medical devices, what they communicate to patients, and their likelihood of reporting safety events associated with medical products.

## Methods

We conducted a survey among a stratified random sample selected from the national population of physicians board certified in internal medicine, cardiovascular medicine, or medical oncology (see the “Methods” section in the supplemental material).^23^ Cardiologists and medical oncologists were included because they most often use newly approved medical devices and drugs, respectively. The American Board of Internal Medicine (ABIM) provided physician contact information.

Among 320,971 physicians included in the ABIM database, physicians were excluded if they did not have active board certification; had inactive medical licenses, addresses outside of the United States, missing variables, or an invalid e-mail address; were retired or deceased, blocked from participation, older than 70 y as of January 2021, board certified and practicing since before 1990, or certified after 2015; or were not internists, cardiologists, or oncologists. This left 59,393 internists, 16,507 cardiologists, and 8,944 oncologists. A random sample of 50 physicians from this stratified random sample were included in a pilot to test distribution. After this pilot, 1,450 physicians were invited to complete the survey.

The study was approved by the institutional review board with voluntary survey completion implying informed consent.

### Survey Instrument

We developed survey questions covering 3 domains: factors influencing physician decisions to prescribe a drug or recommend a medical device, physicians’ perceptions of their communication with patients about new drugs and devices, and physicians’ likelihood of reporting adverse events (Figure A in the supplemental material). These survey questions were developed using key informant interviews,^24^ a modified Delphi panel,^25^ and the authors’ expertise in this area, including both shifts in medical product regulation and multiple prior national survey studies of physicians.^26,27^ We expected most physicians would be familiar with most aspects of the survey questions. However, for survey questions relating to more specialized aspects of FDA regulation about which we did not expect all physicians to necessarily have familiarity (such as the definition of a surrogate measure and historical control as well as the rationale and evidentiary basis for accelerated approval), we provided essential contextual information before the survey question.

#### Factors influencing the decision to recommend a medical product

First, we asked physicians how much various factors influence their decision to use new drugs or devices (answer choices: a lot, somewhat, a little, none). Contributors included relying on FDA approval, clinical trial data, professional resources, interaction with sales representatives, or the practices of trusted colleagues.

#### Communication with patients

Next, we asked about physician-patient communication, including whether physicians mentioned that new drugs or devices were usually advances and whether they discussed the time needed after approval to establish a track record of safety. Later in the survey, after first providing background information on accelerated approval, an expedited pathway that allows FDA approval based on surrogate measures and requires further postmarket study, we asked if patients should be informed that it was unknown if the drug had an effect on a clinical endpoint and if drug promotion should await the generation of confirmatory evidence. We provided a definition of a surrogate measure, stating that it “substitutes for a clinical endpoint,” that “[s]urrogate measures can include biomarkers or other laboratory tests (e.g., hemoglobin A1c level), and changes on imaging (e.g., tumor size),” and that such measures contrast with clinical endpoints, which directly measure “how a patient feels, functions, or survives.” To avoid social desirability bias^28^ and superiority bias,^29^ we randomized respondents 1:1 using the Qualtrics survey tool to either questions asking them to reflect on their own experiences or on their perceptions of average physicians in their specialties. By asking either about respondents’ actions or those of the average physician, we expected to be able to view both results and better understand communication to patients and safety event reporting. We asked physicians what they communicated to patients about the evidence supporting a medical product: the use of expedited approval programs; whether the trials relied on for approval were randomized, had active comparators, or used surrogate versus clinical endpoints; and if there was an ongoing required postapproval trial. Physicians could reply that they usually discussed the given factor, did not consider the factor relevant, or did not have information about the factor.

#### Randomization to understand adverse event reporting

Finally, we asked physicians if they saw a patient who had an adverse event “probably related to a drug or medical device,” how likely (answer choices: very, somewhat, not) they were to report the event to FDA, manufacturer, or health system. Physicians were again randomized to reflect on their own experience or the experience of the average physician in their specialty. We also asked respondents why they may not report the adverse event (answer choices: lack of time, not knowing they should report, not feeling it was their duty to report, not knowing how to report, expecting patients to report).

### Survey Pretesting and Distribution

We piloted the survey on a convenience sample of 11 physicians, revised it based on their feedback, followed with cognitive testing of the revised version with 2 more physicians, then again refined the questions and answer choices. Starting in October 2021, we e-mailed a survey link to the randomly selected physicians, offering a $75 Amazon gift card for completion. We sent up to 7 e-mail reminders, each with an opportunity to opt out. For physicians who had not responded, we mailed a letter with a $10 bill, QR code, and survey link, offering a $65 Amazon gift card honorarium, and followed with up to 4 additional e-mail reminders. We closed the survey on September 27, 2022.

### Statistical Analysis

Analysis was performed based on American Association for Public Opinion Research standards.^30^ We included all partial responses from physicians who replied after e-mail invitations and only the first response if a physician attempted the survey multiple times, and we excluded survey submissions in which the same multiple-choice answer was selected throughout. For questions about adverse event reporting, we combined “very” and “somewhat” likely to present data on whether (“very” or “somewhat”) or not (“not”) physicians stated that they or the average physician in their specialty were likely to report an adverse event. To extract possible differences between responses to questions asking about average physicians versus survey respondents themselves, we used chi-square tests. To check for possible self-selection bias, we compared responses to all nonrandomized questions between physicians who replied to e-mail invitations and those who replied after mailed invitations as well as between the first 50 and final 50 respondents. We also compared available demographic characteristics among physicians who completed the survey to physicians in oncology, cardiology, and internal medicine based on publicly available demographic data from the Association of American Medical Colleges (AAMC) about the current United States physician workforce.^31^ Analyses were performed using SAS 9.4 software (SAS Institute, Cary, NC, USA).

## Results

Of 1,450 invitees, 159 were excluded due to auto-returned e-mails or mail, and 509 responded (39%, with 478 full responses that provided demographic characteristics; see the “Methods” in the supplemental materials). There were 186 internists, 146 cardiologists, and 146 oncologists (Table A in the supplemental material). Although at the time of data analysis we were unable to compare the demographic data of physicians who completed the survey with those of the overall population from which they were sampled, there were no significant differences in age, gender, or geographic region between physicians who were invited and completed the survey versus those who were invited and did not complete the survey. Survey respondents had similar demographic characteristics to the overall current US population of physicians in oncology, cardiology, and internal medicine based on publicly available demographic data from the AAMC (Table B in the supplemental material).

The gender distribution included 312 (65%, 95% confidence interval [CI] 61%–70%) male, 150 (31%, 95% CI 27%–36%) female, 1 (0.2%, 95% CI 0.0%–0.6%) transgender female, 1 (0.2%, 95% CI 0.0%–0.6%) nonbinary, and 14 (3%, 95% CI 1%–4%) who declined to answer. Race and ethnicity data allowed for multiple responses, and the survey included 248 (51%, 95% CI 47%–56%) White respondents; 152 (31%, 95% CI 28%–36%) Asian; 16 (3%, 95% CI 2%–5%) Black or African American; 17 (4%, 95% CI 2%–5%) Hispanic, Latinx, or Spanish origin; 1 (0.2%, 95% CI –0.2% to 0.6%) Pacific Islander; 0 (95% CI 0%–0%) American Indian or Native American; 36 (7%, 95% CI 5%–10%) other; and 15 (3%, 95% CI 2%–5%) who declined to answer. The US geographic location of practice included 113 (24%, 95% CI 20%–27%) in the Northeast, 158 (33%, 95% CI 29%–37%) in the South, 92 (19%, 95% CI 16%–23%) in the Midwest, and 115 (24%, 95% CI 20%–28%) in the West. For years since residency completion, 79 (17%, 95% CI, 13%–20%) reported <10 y, 118 (25%, 95% CI 21%–29%) 10 to 14 y, 98 (21%, 95% CI 17%–24%) 15 to 19 y, and 183 (38%, 95% CI 34%–43%) ≥20 y.

Primary practice areas included 186 (39%, 95% CI 35%–43%) internal medicine, with a distribution of 103 (22%, 95% CI 18%–25%) primary care, 71 (15%, 95% CI 12%–18%) hospital medicine, and 12 (3%, 95% CI 1%–4%) other internal medicine; 146 (31%, 95% CI 26%–35%) oncology; and 146 (31%, 95% CI 26-35%) cardiology, with a distribution of 77 (16%, 95% CI 13%–19%) general cardiology, 23 (5%, 95% CI 3%–7%) cardiac electrophysiology, 40 (8%, 95% CI 6%–11%) interventional cardiology, 5 (1%, 95% CI 0.1%–2%) advanced heart failure, and 2 (0.4%, 95% CI –0.2% to 1%) other cardiology.

### Factors Influencing the Decision to Recommend a Medical Product

**Figure 1:**
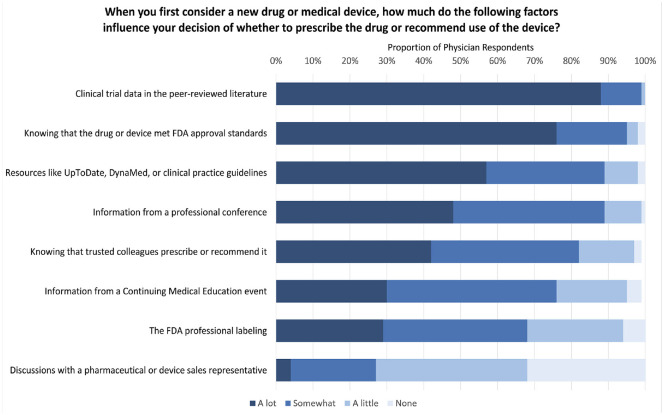
Factors influencing physician decision to prescribe a new drug or recommend medical device. Percentages may not add to 100% due to rounding.

Physicians reported their drug and medical device prescribing was substantially influenced by clinical trial data in the peer-reviewed literature (446, 88%), knowing the drug or device met FDA approval standards (387, 76%), and clinical resources such as UpToDate (388, 57%) (Figure 1). Discussions with a pharmaceutical or device sales representative most often influenced decision making somewhat (23%) or a little (41%). Only 167 (33%) reported no influence from pharmaceutical or medical device sales representatives. Results were overall consistent when comparing physicians who replied after the initial e-mail invitations and those who replied after mailed invitations, as well as comparing the first 50 and final 50 respondents (Figures B and C in the supplemental material).

### Communication with Patients

Most (436, 86%) physicians reported generally telling patients when using a new drug or device that it takes time to establish a track record for safety (Table 1). Many (354, 70%) also reported telling patients that the new drugs/devices were advances compared with previously approved therapies. For drugs approved through accelerated approval, 333 (69%) physicians said patients should definitely be informed that it is unknown if the drug has an effect on a clinical endpoint, while 129 (27%) said patients should probably be informed. Similarly, 420 (87%) thought there definitely or probably should not be drug promotion for accelerated approval drugs until confirmatory evidence was generated. Results were again consistent comparing physicians who replied after the initial e-mail invitations with those who replied after mailed invitations as well as comparing the first 50 and final 50 respondents (Tables C and D in the supplemental material).

**Table 1:** Communication about New Drugs and Devices to Patients^a^

When you are discussing the prescription of a new drug or recommending a new medical device to a patient, do you generally tell the patient that: (*n* = 509), No. (%) answering “yes”	
The drug/device is new	496 (97)
New drugs/devices are usually advances compared to previously approved drugs/devices	354 (70)
It takes time to establish a track record for the safety of new drugs/devices	436 (86)
With respect to drugs approved through the accelerated approval pathway, do you think: (*n* = 483), No. (%)	
Patients being prescribed the drug should be informed that we do not know if the drug has an effect on a clinical endpoint	
Definitely yes	333 (69)
Probably yes	129 (27)
Probably not	17 (4)
Definitely not	4 (1)
There should not be drug promotion until confirmatory clinical evidence is generated	
Definitely yes	253 (52)
Probably yes	167 (35)
Probably not	55 (11)
Definitely not	8 (2)

a Percentages may not add to 100% due to rounding.

#### Randomization results for social desirability bias

Physicians randomized to questions asking about their own discussions with patients consistently reported greater communication of information about expedited approval pathways and limitations in supporting evidence compared with physicians randomized to questions asking about the average physician (Table 2). This finding was generally consistent across the 3 specialties (Tables E–G in the supplemental material). About half (129, 53%) reported usually discussing with patients that a drug or medical device had received expedited approval, compared with 104 (41%) commenting on the average physician (*P* = 0.003).

**Table 2:** Communication about FDA Approval and Strength of Evidence with Patients among Physicians Reporting Their Own Communication and Communication of the Average Physician in their Specialty^a^

Assume ___ are planning to prescribe a new drug or recommend use of a new medical device and are discussing this plan with a patient. Do[es] ___ usually include a discussion of the following factors with the patient?			
	“You”^b^ (*n* = 243), No. (%)	“The Average Physician in Your Specialty” (*n* = 251), No. (%)	*P* Value
It received expedited FDA approval			
Yes	129 (53)	104 (41)	0.003
No: I/the average physician do[es]n’t consider this factor is relevant	84 (35)	89 (35)	
No: I/the average physician do[es]n’t have this information	30 (12)	58 (23)	
It was approved based on a randomized trial			
Yes	182 (75)	146 (58)	<0.001
No: I/the average physician do[es]n’t consider this factor is relevant	46 (19)	73 (29)	
No: I/the average physician do[es]n’t have this information	15 (6)	32 (13)	
It was approved based on a single-arm trial using a historical control			
Yes	85 (35)	41 (16)	<0.001
No: I/the average physician do[es]n’t consider this factor is relevant	105 (43)	111 (44)	
No: I/the average physician do[es]n’t have this information	53 (22)	99 (39)	
It was approved based on showing improvement on a clinical endpoint			
Yes	211 (87)	183 (73)	<0.001
No: I/the average physician do[es]n’t consider this factor is relevant	24 (10)	40 (16)	
No: I/the average physician do[es]n’t have this information	8 (3)	28 (11)	
It was approved based on showing improvement on a surrogate measure			
Yes	122 (50)	81 (32)	<0.001
No: I/the average physician do[es]n’t consider this factor is relevant	77 (32)	113 (45)	
No: I/the average physician do[es]n’t have this information	44 (18)	57 (23)	
There is an ongoing FDA-required postapproval trial to evaluate safety and effectiveness			
Yes	126 (52)	78 (31)	<0.001
No: I/the average physician do[es]n’t consider this factor is relevant	66 (27)	87 (35)	
No: I/the average physician do[es]n’t have this information	51 (21)	86 (34)	

FDA, US Food and Drug Administration.

a Percentages may not add to 100% due to rounding.

b “You” refers to the physician responding to the survey.

About one-third (85, 35%) reported usually communicating to patients when a drug or medical device was approved based on a single-arm trial using a historical control; however, fewer (41, 16%) felt the average physician in their specialty communicated this information (*P* < 0.001). Similarly, 122 (50%) reported usually telling patients when medical products were approved based on surrogate measures, while 77 (32%) reported not considering this factor relevant to communicate; by contrast, only 81 (32%) thought that the average physician in their specialty conveyed this information to patients, and 113 (45%) did not think the average physician considered this factor relevant to communicate (*P* < 0.001).

Finally, 126 (52%) physicians reported usually telling patients when there was a required FDA postapproval trial to evaluate safety and effectiveness; by contrast, 78 (31%) stated that the average physician in their specialties usually shared this information with patients (*P* < 0.001).

### Adverse Event Reporting

Two-thirds (165, 67%) reported being very or somewhat likely to report drug- or device-related adverse events to the FDA; by contrast, only 123 (49%) responded that the average physician in their specialty would report to the FDA (*P* < 0.001) (Table 3). Higher proportions would report to their health systems (85% of respondents reported that they would, compared with 74% reporting that the average physician in their specialty would; *P* = 0.003). When asked why they may not report, the most common reasons were not knowing how (68% of physicians answering for themselves and 91% commenting on the average physician in their specialty, *P* < 0.001), not having time (68% and 89%, respectively, *P* < 0.001), and not knowing they should (55% and 80%, respectively, *P* < 0.001). Findings were generally consistent across all specialties (Tables H–J in the supplemental material).

**Table 3:** Drug or Medical Device Adverse Event Reporting among Physicians Reporting Their Own Actions and Actions of the Average Physician in Their Specialty

	“You”^a^ (*n* = 245), No. (%)	“The Average Physician in Your Specialty” (*n* = 249), No. (%)	*P* Value
If ___ sees a patient who has an adverse event that is probably related to a drug or medical device, how likely is it that ___ will report this adverse event? No. (%) physicians reporting “very likely” or “somewhat likely”^b^			
To the FDA	165 (67)	123 (49)	<0.001
To the manufacturer	151 (62)	118 (47)	0.002
To the health system’s safety reporting system	208 (85)	184 (74)	0.003
What are the reasons for which ___ might not report that adverse event?			
Don’t have time to report	167 (68)	222 (89)	<0.001
Didn’t know that I/they should report	135 (55)	200 (80)	<0.001
Do not feel it is my/their duty to report	33 (13)	117 (47)	<0.001
Don’t know how to report	166 (68)	226 (91)	<0.001
Expect patients to report	19 (8)	23 (9)	0.55

FDA, US Food and Drug Administration.

a “You” refers to the physician responding to the survey.

b Overall, 81 (33%) of physician respondents stated that they were very likely to report to the FDA, 65 (27%) to the manufacturer, and 129 (53%) to the health system’s safety reporting system; 22 (9%) of physician respondents stated that the average physician in their specialty was very likely to report to the FDA, 32 (13%) to the manufacturer, and 76 (31%) to the health system’s safety reporting system.

## Discussion

In a national survey, board-certified physicians in 3 specialties reported that they prominently relied on FDA approval status when deciding whether to use a drug or device in clinical practice and reported they did not tend to discuss with patients some factors associated with key limitations in the evidence that supported FDA approval. A substantial proportion of physicians also stated that they would not report to the FDA adverse events associated with these drugs or medical devices. These findings suggest a need for strategies to better inform physicians about the evidence supporting FDA approval, ensure more complete communication of this evidence to patients, and promote physician reporting of drug- and device-related adverse events. Such steps can improve understanding of the safety and effectiveness of new drugs and medical devices to support patients in making informed care choices.

It is reassuring that most physicians reported consulting the peer-reviewed literature when considering whether to use a new drug or medical device in clinical practice. However, even when consulted, the peer-reviewed literature may be affected by publication bias in which not all clinical trials for FDA-regulated products are published.^32,33^ Delays between trial completion and publication can also limit available information.^34–36^ Further, peer-reviewed publications of drug and device trials are often funded by industry and may selectively report positive findings.^37–39^

We found that in recommending new treatments, physicians reported that they often place great weight on the fact that products received FDA authorization. However, physicians may be unaware of the variable evidence used in the FDA’s process^24,25^ and in particular the fact that an increasing number of drugs and devices come to market using expedited programs that leave more uncertainties as to clinical benefits, which can be due to reliance on surrogate measures as primary trial endpoints.^2–5^ Other studies have found that physicians tend to overestimate benefits and underestimate harms of treatments,^40^ and most consider drugs that come to market through an expedited pathway to be more effective than they actually are.^26^

Most physicians also reported that discussions with a pharmaceutical or device sales representative influenced their decision making at least a little when they first considered a new drug or medical device, and the responses of the one-third of physicians reporting no influence from sales representatives could be the result of lack of contact with representatives, lack of susceptibility to their influence, or answers that reflect social desirability bias. These findings are consistent with other research finding that half of primary care practices received weekly detailing visits from pharmaceutical companies.^41^ Industry ties can be stronger for specialists who routinely use medical devices as part of procedural interventions because medical device representatives may be present during surgery or procedures for their expertise,^42^ sales/service factors may influence the choice of medical device vendor,^43,44^ and patients may be less involved in product selection. Alternative mechanisms, such as academic detailing^45^ and improved labeling,^46^ could be used to provide physicians with unbiased and up-to-date information about medical products.

The rates of reporting adverse events to the FDA and manufacturers conveyed by physicians in our study—in which the average physician was thought to report less often than physicians answering for themselves—are concerning because the lack of awareness of associated adverse events could make drugs and medical devices appear safer than they actually are and preclude accurate benefit/risk determinations. Most physicians stated that they or the average physician in their specialty would report to their hospital’s safety reporting system; however, a Department of Health and Human Services Office of Inspector General report found that hospital staff did not report 86% of events to incident reporting systems.^47^ Although spontaneous adverse event reporting has many limitations and generally cannot establish causation, it is the information source that most commonly catalyzes drug and medical device safety updates.^48,49^ Our findings suggest that there is a need for new strategies to best support incident reporting. These could include medical education helping physicians understand their duty to report and providing information about how to report. Linkage through electronic health records or a mobile application could facilitate reporting and complement the development of rigorous methods for active safety surveillance.^50^

Consistent with other surveys of how physicians perceive their own practices versus the profession in general,^51^ most reported that they were personally more likely to share information with patients compared with the average physician in their specialty. Following the social psychology concept of superiority bias (a tendency of individuals to overestimate their abilities or performance relative to others),^29^ we found that physicians are either overestimating the depth of their communication with patients or underestimating their colleagues’ communication. Prior research analyzing recordings of physician-patient conversations has demonstrated that physicians may minimize risks and emphasize benefits for novel drugs and for device-based procedures.^52,53^ This suggests physicians in our survey may have overestimated their own reporting.

If communication to patients is being overestimated, it is concerning that fewer than half of physicians reported conveying to patients when a drug was approved based on improvement in a surrogate measure and at least one-third did not even consider this factor relevant. The use of surrogate measures means that patients cannot be assured of any clinical outcome benefit.^6^ Confusion over expected benefits can be worsened when such drugs or medical devices are simultaneously granted FDA’s “Breakthrough” designation.^26,54,55^ The “Breakthrough” designation is intended to expedite the development of a drug or medical device that provides for more effective diagnosis or treatment of serious conditions, such as through the engagement of senior FDA personnel and priority FDA review. However, FDA’s guidance for breakthrough devices includes the possibility of approvals based on “a greater extent of uncertainty of the benefit-risk profile.”^9^ If patients are not informed about the certainty and magnitude of benefit, they may overestimate those benefits^56^; indeed, research has found that use of the word “breakthrough” related to a drug leads physicians and patients to consider the drug as more effective than it actually is based on the supporting evidence.^26,55^

Despite the legal requirement to “verify . . . clinical benefit” in postapproval studies,^57^ drugs may not have their clinical outcome benefits verified^18^ or may even be shown to have no clinical outcome benefit and yet not be immediately removed from the market.^58,59^ Postmarket evidence generation is not only required for drugs approved through the accelerated approval program but is also frequently required by FDA as a condition of approval for drugs and devices approved via traditional pathways.^60,61^ Although physicians reported appropriately advising patients that it takes time to establish a track record of safety, we found that the continuing need for postmarket evidence to fill in evidence gaps is often not conveyed to patients, who may be vulnerable to novelty bias—a tendency to perceive “new” as better.^62^

Our study has multiple limitations. Our sample was limited to internists, cardiologists, and oncologists and may not generalize to other specialties. We did not ask physicians about multiple factors that may have affected their responses, including their practice setting, practice size, time per patient encounter, and experience prescribing/ordering novel drugs and medical devices. Despite our attempts to reduce bias through an anonymous survey, superiority and social desirability bias could have affected responses. It is also possible that the approximately 40% of physicians who responded to the survey differ from nonparticipants and are more likely to communicate information that we asked about to patients. In addition, some questions, such as asking physicians whether they tell patients that new drugs/devices are advances compared with previously approved drugs/devices or if they discuss that it takes time to establish a track record of safety for new drugs/devices, could have suggested or led participant responses.

Despite these limitations, physicians’ self-reported communication practices suggest that they sometimes do not discuss important aspects of the evidence supporting newly approved drugs and medical devices with patients, particularly when these products come to market using expedited programs or based on surrogate measures. Safety concerns may be underrepresented due to lack of physician reporting of adverse events. Reforms such as greater clarity in drug and medical device labeling, sensible regulation of advertising, the teaching of FDA regulatory standards in medical education, and the reduction of conflicts of interest in medicine are needed to ensure that physicians receive more unbiased information and convey it faithfully to patients.

## Supplemental Material

sj-docx-1-mdm-10.1177_0272989X241302096 – Supplemental material for Physician-Patient Communication about Novel Drugs and High-Risk Medical Devices Supplemental material, sj-docx-1-mdm-10.1177_0272989X241302096 for Physician-Patient Communication about Novel Drugs and High-Risk Medical Devices by Sanket S. Dhruva, Aaron S. Kesselheim, Steven Woloshin, Robin Z. Ji, Zhigang Lu, Jonathan J. Darrow and Rita F. Redberg in Medical Decision Making
