# Supplementary material for: Physician-Patient Communication about Novel Drugs and High-Risk Medical Devices
Source: Med Decis Making. 2024 Dec 21;45(2):156–67. doi: 10.1177/0272989X241302096 (PMC11736971; doi:10.1177/0272989X241302096)
Supplement: sj-docx-1-mdm-10.1177_0272989X241302096 – Supplemental material for Physician-Patient Communication about Novel Drugs and High-Risk Medical Devices [file sj-docx-1-mdm-10.1177_0272989X241302096.docx]

**Online-Only Supplemental Material for Physician-Patient Communication about Novel Drugs and Medical Devices**

| **Table of Contents** | Page |
| --- | --- |
| Methods. Description of Stratified Random Sample, Invitation and Survey Process, and Respondent Characteristics | 31 |
| Figure A. Content of Survey Sent to Physicians | 32 |
| Figure B. Factors Influencing Physician Decision to Prescribe New Drug or Recommend Medical Device, Email vs. Mail Respondents | 51 |
| Figure C. Factors Influencing Physician Decision to Prescribe New Drug or Recommend Medical Device, First 50 vs. Last 50 Respondents | 52 |
| Table A. Characteristics of Physician Survey Respondents | 53 |
| Table B. Comparison of Physician Survey Respondents to Current Physician Workforce using Association of American Medical Colleges Data | 55 |
| Table C. Communication about New Drugs and Devices to Patients, Email vs. Mail Respondents | 56 |
| Table D. Communication about New Drugs and Devices to Patients, First 50 vs. Last 50 Respondents | 57 |
| Table E. Discussion of Approval Factors, Internists | 58 |
| Table F. Discussion of Approval Factors, Cardiologists | 60 |
| Table G. Discussion of Approval Factors, Oncologists | 62 |
| Table H. Drug or Medical Device Adverse Event Reporting Behavior, Internists | 64 |
| Table I. Drug or Medical Device Adverse Event Reporting Behavior, Cardiologists | 65 |
| Table J. Drug or Medical Device Adverse Event Reporting Behavior, Oncologists | 66 |

**Methods. Description of Stratified Random Sample, Invitation and Survey Process, and Respondent Characteristics**

*Power Calculation*

We estimated the number of physicians who we would need to survey based partially on a similar survey question that members of our research team had previously asked to physicians and published in the peer-reviewed literature and a hypothetical question that we conceived and planned to ask physicians: “On a Likert scale of 1-5, when using a novel drug/device/digital health technology, how likely are you to convey uncertainty of benefit and risk?” (1 = unlikely and 5 = very likely, with a binary factor of 3+ meaning uncertainty is conveyed and 1-2 meaning it is not). We anticipated that 7% of physicians would convey uncertainty (answers 3 -5) with a result that would have p-value <0.05. Accordingly, we estimated that we would need 674 physicians – an approximately 46% response rate from surveying 1450 physicians.

*Construction of Stratified Random Sample*

A total of 320,971 physicians were included in the database. Of these, 22,092 were deceased or retired; 20,980 had inactive licenses; 1441 were blocked from participation; 25,613 were over the age of 70 as of January 2021; 37,657 were Board-certified and practicing since before 1990; 42,980 were certified after 2015; and 57,270 were not categorized as general internists, cardiologists, or oncologists. All of these physicians were excluded. This left 112,758 general internists, cardiologists, and oncologists for inclusion. Of this group, a further 19,119 were without currently active certification, 1127 had addresses outside of the United States, 465 had missing variables, 230 had an invalid email address, and 6973 were missing residency or fellowship information. After excluding those physicians, a total 84,844 physicians remained for a stratified random sample of 500 each from: 59,393 general internists, 16,507 cardiologists, and 8944 oncologists. A random sample of 50 physicians from this stratified random sample were included in a pilot to test distribution.

*Invitation and Survey Process*

A total of 1450 American Board of Internal Medicine (ABIM) diplomates (not included in pilot test distribution) were sent an email invite. Of these, 22 were excluded because they did not receive the email invitation due to a bounceback; this left 1428 who received an email invite. Of these 1428, 9 opted out of the survey. Three hundred fifty-two physicians responded to the survey via email invite within 8 total invites (1 initial email and 7 reminders). Overall, 1098 ABIM diplomates were sent a letter invite; of these, 137 were excluded as they did not receive the letter invitation due to bounceback. This left 961 physicians who received a letter invite, 184 of whom responded to the survey after mailing of this letter. Overall, there were a total of 536 total responses at this stage. Of these, 12 were excluded because they were duplicate responses following an initial response, 13 were excluded as they were partial responses resulting from a letter invite, and 2 were excluded as they were responses with homogeneity across column questions, resulting in 509 total included responses. Four hundred seventy-eight of the 509 responding physicians completed the entire survey and provided demographic characteristics, including 186 internists, 146 cardiologists, and 146 oncologists.

**Figure A. Content of Survey Sent to Physicians**

Start of Block: Intro

**Physicians’ Perspectives on FDA Approvals Survey (PPFAS)**

**UCSF - Harvard - Dartmouth Survey on New Drugs & Medical Devices**

Dear Colleague,

Not another survey! 
 
We know you’re busy and get asked to complete a lot of surveys. Before you trash this, please take a minute to read on about why you might care about this survey.
 
**What is this about?** The FDA has been in the news a lot lately - we want to know your thoughts and views about FDA approval for new drugs and medical devices. 
 
**Why you?**  You were randomly selected as part of a national sample of physicians identified via ABIM. All survey responses will be kept strictly confidential and will not be linked to your name.
 
**Who is responsible?** The survey is funded by The Greenwall Foundation, a tax-exempt 501(c)(3) organization, and conducted by researchers at the University of California, San Francisco (UCSF), Harvard Medical School/Brigham and Women’s Hospital, and Dartmouth Institute for Health Policy and Clinical Practice. The study has been approved by the Institutional Review Board of UCSF. 
 
**What are we asking you to do?** Please complete the survey in one sitting (which should take about 15 minutes). Upon completion, you will receive your Amazon gift card credit. If you have any questions, please e-mail us at greenwallfoundationsurvey@ucsf.edu.  
 
Please try to complete the survey in one sitting, although you can resume later if necessary. You will need to answer all questions on a page before moving to the next page. 
 
Thank you very much.

End of Block: Intro

Start of Block: Section 1: FDA Approval of New Drugs and Devices

**FDA Approval of New Drugs and Devices**
 
**Our first few questions will ask how you think about new drugs or medical devices.**


**By "new drug," we mean a brand-name drug recently approved by the FDA in the United States.  By "new medical device," we are focused on the Premarket Approval pathway, which is one of the main ways that medical devices are reviewed by the FDA.  This pathway is used for new high-risk medical devices, many of which are implanted, such as implantable biosensors for cancer treatment, pacemakers, transcatheter aortic valves, and deep brain stimulators.**


**When answering the questions, please consider new drugs or medical devices for conditions that you commonly manage.**

When you first consider a new drug or medical device, how much do the following factors influence your decision of whether to prescribe the drug or recommend use of the device?

|  | A lot | Somewhat | A little | None |
| --- | --- | --- | --- | --- |
| Knowing that the drug or device met FDA approval standards |  |  |  |  |
| Clinical trial data in the peer-reviewed literature |  |  |  |  |
| Resources like UpToDate, DynaMed, or clinical practice guidelines |  |  |  |  |
| The FDA professional labeling (i.e., the paper insert with detailed drug information or instructions/directions for use) |  |  |  |  |
| Discussions with a pharmaceutical or device sales representative |  |  |  |  |
| Information from a Continuing Medical Education event |  |  |  |  |
| Information from a professional conference |  |  |  |  |
| Knowing that trusted colleagues prescribe or recommend it |  |  |  |  |

When you are discussing the prescription of a new drug or recommending a new medical device to a patient, do you generally tell the patient that:

|  | Yes | No |
| --- | --- | --- |
| The drug/device is new |  |  |
| New drugs/devices are usually advances compared to previously-approved drugs/devices |  |  |
| It takes time to establish a track record for the safety of new drugs/devices |  |  |

End of Block: Section 1: FDA Approval of New Drugs and Devices

**At this point in the survey, the survey respondents were randomized into two groups. The first group was given the 5 questions under the block titled “yourself questions” while the second group was given the 5 questions under the block titled “other physician questions”. The two groups were rejoined in the section afterwards, titled “Section 2: New Drugs”.**

Start of Block: yourself questions

Assume you are planning to prescribe a new drug or recommend use of a new medical device and are discussing this plan with your patient. Do you usually include a discussion of the following factors with the patient?

|  |  | | |
| --- | --- | --- | --- |
|  | Yes | No - I don't consider this factor is relevant | No - I don't have this information |
| It received **expedited** FDA approval |  |  |  |
| It was approved based on a **randomized** trial |  |  |  |
| It was approved based on a **single-arm** trial using a historical control |  |  |  |
| It was approved based on showing improvement on a **clinical endpoint** |  |  |  |
| It was approved based only on showing improvement on a **surrogate measure** |  |  |  |
| There is an ongoing FDA required **post-approval trial** to evaluate safety and effectiveness |  |  |  |

**Now, we are going to shift and ask questions about adverse events, defined as undesirable experiences associated with the use of a drug or medical device in a patient.**

If you see a patient who has an adverse event that is probably related to a drug or medical device, **how likely is it that you will report** this adverse event:

|  | Very likely | Somewhat likely | Not likely |
| --- | --- | --- | --- |
| To the FDA? |  |  |  |
| To the manufacturer? |  |  |  |
| To your health system's safety reporting system? |  |  |  |

What are the reasons for which you might not report that adverse event:

|  | Yes | No |
| --- | --- | --- |
| I don’t have time to report |  |  |
| I didn't know that I should report |  |  |
| I do not feel it is not my duty to report |  |  |
| I don’t know how to report |  |  |
| I expect patients to report |  |  |

Would you answer differently if the patient experienced a medical device-related adverse event compared to a drug-related adverse event?

- Yes
- No

Please explain how and why there would be a difference in your reporting a drug- or device-related adverse event. (**Optional**, if you have thoughts)

________________________________________________________________

End of Block: yourself questions

Start of Block: other physician questions

|  |
| --- |

We would now like to ask what you think **the average physician in your specialty discusses with patients.**

When physicians are planning to prescribe a new drug or recommend use of a new medical device and are discussing this plan with the patient, do they usually include a discussion of the following factors with the patient?

|  |  | | |
| --- | --- | --- | --- |
|  | Yes | No - the average physician doesn't consider this factor relevant | No - the average physician doesn't have this information |
| It received **expedited** FDA approval |  |  |  |
| It was approved based on a **randomized** trial |  |  |  |
| It was approved based on a **single-arm** trial using a historical control |  |  |  |
| It was approved based on showing improvement on a **clinical endpoint** |  |  |  |
| It was approved based only on showing improvement on a **surrogate measure** |  |  |  |
| There is an ongoing FDA required **post-approval trial** to evaluate safety and effectiveness |  |  |  |

**Now, we are going to shift and ask questions about adverse events, defined as undesirable experiences associated with the use of a drug or medical device in a patient.**

If the average physician in your specialty sees a patient who has an adverse event that is probably related to a drug or medical device, **how likely is it that the physician will report** this adverse event:

|  | Very likely | Somewhat likely | Not likely |
| --- | --- | --- | --- |
| To the FDA? |  |  |  |
| To the manufacturer? |  |  |  |
| To their health system's safety reporting system? |  |  |  |

What are the reasons for which the average physician might not report that adverse event:

|  | Yes | No |
| --- | --- | --- |
| Lack of time to report |  |  |
| Did not know he/she/they should report |  |  |
| Do not feel it is his/her/their duty to report |  |  |
| Do not know how to report |  |  |
| Expect patients to report |  |  |

Do you think there are differences if the patient experienced a medical device-related adverse event compared to a drug-related adverse event?

- Yes
- No

Please explain how and why there would be a difference in reporting a drug- or device-related adverse event. (**Optional**, if you have thoughts)

________________________________________________________________

End of Block: other physician questions

Start of Block: Section 2: New Drugs

**FDA Approval of New Drugs**

**Our next questions will specifically ask how you think about new drugs.  As a reminder, by "new drug," we mean a brand-name drug recently approved by the FDA in the United States.**

**Accelerated Approval of New Drugs**

**One way to speed the availability of promising new drugs is via the accelerated approval pathway, which allows FDA approval based on surrogate measures that are "reasonably likely" to predict meaningful clinical endpoints. Accelerated approval drugs are required to undergo confirmatory clinical trials after approval.**  

With respect to drugs approved through the accelerated approval pathway, do you think:

|  | Definitely yes | Probably yes | Probably not | Definitely not |
| --- | --- | --- | --- | --- |
| Patients being prescribed the drug should be informed that we do not know if the drug has an effect on a clinical endpoint |  |  |  |  |
| There should not be drug promotion until confirmatory clinical evidence is generated |  |  |  |  |

End of Block: Section 2: New Drugs

Start of Block: Final Section: Basic Professional and Demographic Characteristics

**This final series of questions relates to professional and demographic characteristics.**

My primary practice area is: (select one)

- Primary Care
- Hospital Medicine
- Oncology
- General Cardiology
- Cardiac Electrophysiology
- Interventional Cardiology
- Advanced Heart Failure
- Other (Please specify) ________________________________________________

Please enter the 5-digit U.S. ZIP code for the primary location in which you practice. By primary practice we mean the place where you spend the majority of your clinical time.

________________________________________________________________

Which term do you use to describe your current gender identity?

- Male
- Female
- Non-binary
- Transgender male
- Transgender female
- Other
- Prefer not to answer

What is your racial or ethnic identification? (Select as many as apply):

- American Indian or Native American
- Asian
- Black or African American
- Hispanic, Latinx, or of Spanish origin
- Pacific Islander
- White
- Other (Please specify)
- Prefer not to answer

In what year did you complete residency? (enter 4-digit year)

________________________________________________________________

In the last 24 months, have you received **any research-related payments** from a drug, device, or other medically-related company?

- Yes
- No

In the last 24 months, have you received any **non-research-related payments** (e.g. meals, consulting arrangements, free or subsidized admission to meetings or conferences) from a drug, device, or other health-related company?

- Yes
- No

End of Block: Final Section: Basic Professional and Demographic Characteristics

**Figure B. Factors Influencing Physician Decision to Prescribe New Drug or Recommend Medical Device, Email vs. Mail Respondents**

^a^Statistically significant difference, p= 0.03.

**Figure C. Factors Influencing Physician Decision to Prescribe New Drug or Recommend Medical Device, First 50 vs. Last 50 Respondents**

^a^Statistically significant difference, p= 0.01.

**Table A. Characteristics of Physician Survey Respondents**

|  | **All Respondents (N=478), n** | **Internal Medicine**  **(N = 186), n** | **Cardiology**  **(N = 146), n** | **Oncology**  **(N = 146), n** |
| --- | --- | --- | --- | --- |
| **Gender** | | |  |  |
| Male | 312 | 104 | 116 | 92 |
| Female | 150 | 75 | 27 | 48 |
| Transgender female | 1 | 1 | 0 | 0 |
| Non-binary | 1 | 0 | 0 | 1 |
| Declined to answer | 14 | 6 | 3 | 5 |
| **Race/ethnicity (respondents could select multiple responses)** | | |  |  |
| White | 248 | 99 | 76 | 73 |
| Asian | 152 | 53 | 48 | 51 |
| Black or African American | 16 | 8 | 3 | 5 |
| Hispanic, Latinx, or of Spanish Origin | 17 | 9 | 3 | 5 |
| Pacific Islander | 1 | 0 | 0 | 1 |
| American Indian or Native American | 0 | 0 | 0 | 0 |
| Other | 36 | 15 | 11 | 10 |
| Declined to Answer | 15 | 5 | 6 | 4 |
| **Geographic location of practice in the United States** | | |  |  |
| Northeast | 113 | 40 | 36 | 37 |
| South | 158 | 64 | 41 | 53 |
| Midwest | 92 | 39 | 31 | 22 |
| West | 115 | 43 | 38 | 34 |
| **Years since residency completion** | | |  |  |
| <10 | 79 | 27 | 22 | 30 |
| 10-14 | 118 | 37 | 45 | 36 |
| 15-19 | 98 | 29 | 32 | 37 |
| ≥20 | 183 | 93 | 47 | 43 |
| **Primary practice area^a^** | | |  |  |
| Internal medicine | 186 | 186 | 0 | 0 |
| Primary care | 103 | 103 | 0 | 0 |
| Hospital medicine | 71 | 71 | 0 | 0 |
| Other internal medicine | 12 | 12 | 0 | 0 |
| Oncology | 146 | 0 | 0 | 146 |
| Cardiology | 146 | 0 | 146 | 0 |
| General cardiology | 77 | 0 | 77 | 0 |
| Cardiac electrophysiology | 23 | 0 | 23 | 0 |
| Interventional cardiology | 40 | 0 | 40 | 0 |
| Advanced heart failure | 5 | 0 | 5 | 0 |
| Other cardiology | 2 | 0 | 2 | 0 |
| **Payments received** | | |  |  |
| Any payments | 162 | 27 | 57 | 78 |
| Research-related payments | 65 | 7 | 15 | 43 |
| Non research-related payments (e.g., meals, consulting arrangements, free or subsidized admission to meetings or conferences) | 133 | 22 | 51 | 60 |

^a^Physicians who did not select one of the above primary practice areas are classified as “other.”

**Table B. Comparison of Physician Survey Respondents to Current Physician Workforce using Association of American Medical Colleges Data**

| **Demographic Characteristic** | **Physician Survey Respondents** | **AAMC Population^a^** |
| --- | --- | --- |
| Gender, % Women | 31 | 35 |
|  |  |  |
| Race, % Asian | 31 | 25 |
| Race, % Black or African-American | 3 | 6 |
| Race, % white | 51 | 47 |
|  |  |  |
| Ethnicity, % Hispanic or Latino | 4 | 6 |

^a^AAMC Population refers to physicians demographic data from the Association of American Medical Colleges (AAMC) about the current United States physician workforce and limited to physicians in oncology, cardiology, and internal medicine

**Table C. Communication about New Drugs and Devices to Patients, Email vs. Mail Respondents**

| **When you are discussing the prescription of a new drug or recommending a new medical device to a patient, do you generally tell the patient that: No. (%) Answering Yes** | | | |
| --- | --- | --- | --- |
|  | **Email**  **(N = 351)** | **Mail**  **(N = 158)** | **P-value** |
| The drug/device is new | 340 (97) | 156 (99) | 0.22 |
| New drugs/devices are usually advances compared to previously-approved drugs/devices | 241 (69) | 113 (72) | 0.52 |
| It takes time to establish a track record for the safety of new drugs/devices | 306 (87) | 130 (82) | 0.14 |
| **With respect to drugs approved through the accelerated approval pathway, do you think: No. (%)** | | | |
|  | **Email**  **(N = 325)** | **Mail**  **(N = 158)** | **P-value** |
| Patients being prescribed the drug should be informed that we do not know if the drug has an effect on a clinical endpoint | | | |
| Definitely yes | 222 (68) | 111 (70) | 0.44 |
| Probably yes | 91 (28) | 38 (24) |  |
| Probably not / Definitely not | 12 (4) | 9 (6) |  |
| There should not be drug promotion until confirmatory clinical evidence is generated | | | |
| Definitely yes | 178 (55) | 75 (47) | 0.25 |
| Probably yes | 109 (34) | 58 (37) |  |
| Probably not / Definitely not | 38 (12) | 25 (16) |  |

Note: Percentages may not add to 100% due to rounding.

**Table D. Communication about New Drugs and Devices to Patients, First 50 vs. Last 50 Respondents**

| **When you are discussing the prescription of a new drug or recommending a new medical device to a patient, do you generally tell the patient that: No. (%) Answering Yes** | | | |
| --- | --- | --- | --- |
|  | **First 50**  **(N = 50)** | **Last 50**  **(N = 50)** | **P-value** |
| The drug/device is new | 49 (98) | 48 (96) | 0.25 |
| New drugs/devices are usually advances compared to previously-approved drugs/devices | 36 (72) | 38 (76) | 0. 65 |
| It takes time to establish a track record for the safety of new drugs/devices | 45 (90) | 41 (82) | 0.56 |
| **With respect to drugs approved through the accelerated approval pathway, do you think: No. (%)** | | | |
|  | **First 50**  **(N = 47)** | **Last 50**  **(N = 50)** | **P-value** |
| Patients being prescribed the drug should be informed that we do not know if the drug has an effect on a clinical endpoint | | | |
| Definitely yes | 34 (72) | 33 (66) | 0.69 |
| Probably yes | 11 (23) | 13 (26) |  |
| Probably not / Definitely not | 2 (4) | 4 (8) |  |
| There should not be drug promotion until confirmatory clinical evidence is generated | | | |
| Definitely yes | 25 (53) | 18 (36) | 0.14 |
| Probably yes | 19 (40) | 24 (48) |  |
| Probably not / Definitely not | 3 (6) | 8 (16) |  |

Note: Percentages may not add to 100% due to rounding. The number of respondents is fewer than 50 since a small number of respondents had stopped answering the survey before reaching these questions.

**Table E.** **Discussion of Approval Factors, Internists**

| **Assume ___ are planning to prescribe a new drug or recommend use of a new medical device and are discussing this plan with ­­a patient. Do[es] ___ usually include a discussion of the following factors with the patient?** | | | |
| --- | --- | --- | --- |
|  | **“You”^a^ (N = 98),**  **No. (%)** | **“The average physician in your specialty” (N = 88),**  **No. (%)** | **P-value** |
| **It received expedited FDA approval** | | | |
| Yes | 66 (67) | 50 (57) | 0.14 |
| No – I / the average physician do[es]n’t consider this factor is relevant | 19 (19) | 19 (22) |  |
| No – I / the average physician do[es]n’t have this information | 13 (13) | 19 (22) |  |
| **It was approved based on a randomized trial** | | | |
| Yes | 69 (70) | 45 (51) | 0.007 |
| No – I / the average physician do[es]n’t consider this factor is relevant | 18 (18) | 25 (28) |  |
| No – I / the average physician do[es]n’t have this information | 11 (11) | 18 (20) |  |
| **It was approved based on a single-arm trial using a historical control** | | | |
| Yes | 27 (27) | 13 (15) | 0.03 |
| No – I / the average physician do[es]n’t consider this factor is relevant | 38 (39) | 26 (30) |  |
| No – I / the average physician do[es]n’t have this information | 33 (34) | 49 (56) |  |
| **It was approved based on showing improvement on a clinical endpoint** | | | |
| Yes | 81 (83) | 62 (70) | 0.05 |
| No – I / the average physician do[es]n’t consider this factor is relevant | 10 (10) | 12 (14) |  |
| No – I / the average physician do[es]n’t have this information | 7 (7) | 14 (16) |  |
| **Assume ___ are planning to prescribe a new drug or recommend use of a new medical device and are discussing this plan with a patient. Do[es] ___ usually include a discussion of the following factors with the patient?** | | | |
|  | **“You”^a^ (N = 98),**  **No. (%)** | **“The average physician in your specialty” (N = 88),**  **No. (%)** | **P-value** |
| **It was approved based on showing improvement on a surrogate measure** | | | |
| Yes | 52 (53) | 29 (33) | 0.006 |
| No – I / the average physician do[es]n’t consider this factor is relevant | 24 (24) | 32 (36) |  |
| No – I / the average physician do[es]n’t have this information | 22 (22) | 27 (31) |  |
| **There is an ongoing FDA required post-approval trial to evaluate safety and effectiveness** | | | |
| Yes | 59 (60) | 36 (41) | 0.009 |
| No – I / the average physician do[es]n’t consider this factor is relevant | 13 (13) | 18 (20) |  |
| No – I / the average physician do[es]n’t have this information | 26 (27) | 34 (39) |  |

^a^You: referring to the physician responding to the survey

Note: Percentages may not add to 100% due to rounding.

**Table F.** **Discussion of Approval Factors, Cardiologists**

| **Assume ___ are planning to prescribe a new drug or recommend use of a new medical device and are discussing this plan with ­­a patient. Do[es] ___ usually include a discussion of the following factors with the patient?** | | | |
| --- | --- | --- | --- |
|  | **“You”^a^ (N = 69),**  **No. (%)** | **“The average physician in your specialty” (N = 77),**  **No. (%)** | **P-value** |
| **It received expedited FDA approval** | | | |
| Yes | 30 (43) | 22 (29) | 0.06 |
| No – I / the average physician do[es]n’t consider this factor is relevant | 29 (42) | 31 (40) |  |
| No – I / the average physician do[es]n’t have this information | 10 (13) | 24 (31) |  |
| **It was approved based on a randomized trial** | | | |
| Yes | 56 (81) | 46 (60) | 0.005 |
| No – I / the average physician do[es]n’t consider this factor is relevant | 11 (16) | 23 (30) |  |
| No – I / the average physician do[es]n’t have this information | 2 (3) | 8 (10) |  |
| **It was approved based on a single-arm trial using a historical control** | | | |
| Yes | 18 (26) | 8 (10) | 0.01 |
| No – I / the average physician do[es]n’t consider this factor is relevant | 37 (54) | 37 (48) |  |
| No – I / the average physician do[es]n’t have this information | 14 (20) | 32 (42) |  |
| **It was approved based on showing improvement on a clinical endpoint** | | | |
| Yes | 65 (94) | 58 (75) | 0.002 |
| No – I / the average physician do[es]n’t consider this factor is relevant | 4 (6) | 13 (17) |  |
| No – I / the average physician do[es]n’t have this information | 0 (0) | 6 (8) |  |
| **Assume ___ are planning to prescribe a new drug or recommend use of a new medical device and are discussing this plan with a patient. Do[es] ___ usually include a discussion of the following factors with the patient?** | | | |
|  | **“You”^a^ (N = 69),**  **No. (%)** | **“The average physician in your specialty” (N = 77),**  **No. (%)** | **P-value** |
| **It was approved based on showing improvement on a surrogate measure** | | | |
| Yes | 31 (45) | 24 (31) | 0.09 |
| No – I / the average physician do[es]n’t consider this factor is relevant | 26 (38) | 39 (51) |  |
| No – I / the average physician do[es]n’t have this information | 12 (17) | 14 (18) |  |
| **There is an ongoing FDA required post-approval trial to evaluate safety and effectiveness** | | | |
| Yes | 32 (46) | 25 (32) | 0.09 |
| No – I / the average physician do[es]n’t consider this factor is relevant | 26 (38) | 26 (34) |  |
| No – I / the average physician do[es]n’t have this information | 11 (16) | 26 (34) |  |

^a^You: referring to the physician responding to the survey

Note: Percentages may not add to 100% due to rounding.

**Table G.** **Discussion of Approval Factors, Oncologists**

| **Assume ___ are planning to prescribe a new drug or recommend use of a new medical device and are discussing this plan with ­­a patient. Do[es] ___ usually include a discussion of the following factors with the patient?** | | | |
| --- | --- | --- | --- |
|  | **“You”^a^ (N = 70),**  **No. (%)** | **“The average physician in your specialty” (N = 76),**  **No. (%)** | **P-value** |
| **It received expedited FDA approval** | | | |
| Yes | 32 (46) | 27 (36) | 0.21 |
| No – I / the average physician do[es]n’t consider this factor is relevant | 33 (47) | 37 (49) |  |
| No – I / the average physician do[es]n’t have this information | 5 (7) | 12 (16) |  |
| **It was approved based on a randomized trial** | | | |
| Yes | 54 (77) | 49 (64) | 0.09 |
| No – I / the average physician do[es]n’t consider this factor is relevant | 15 (21) | 23 (30) |  |
| No – I / the average physician do[es]n’t have this information | 1 (1) | 4 (5) |  |
| **It was approved based on a single-arm trial using a historical control** | | | |
| Yes | 40 (57) | 20 (26) | <0.001 |
| No – I / the average physician do[es]n’t consider this factor is relevant | 27 (39) | 42 (55) |  |
| No – I / the average physician do[es]n’t have this information | 3 (4) | 14 (18) |  |
| **It was approved based on showing improvement on a clinical endpoint** | | | |
| Yes | 61 (87) | 54 (71) | 0.02 |
| No – I / the average physician do[es]n’t consider this factor is relevant | 8 (11) | 15 (20) |  |
| No – I / the average physician do[es]n’t have this information | 1 (1) | 7 (9) |  |
| **Assume ___ are planning to prescribe a new drug or recommend use of a new medical device and are discussing this plan with a patient. Do[es] ___ usually include a discussion of the following factors with the patient?** | | | |
|  | **“You”^a^ (N = 70),**  **No. (%)** | **“The average physician in your specialty” (N = 76),**  **No. (%)** | **P-value** |
| **It was approved based on showing improvement on a surrogate measure** | | | |
| Yes | 38 (54) | 24 (32) | 0.006 |
| No – I / the average physician do[es]n’t consider this factor is relevant | 24 (34) | 38 (50) |  |
| No – I / the average physician do[es]n’t have this information | 8 (11) | 14 (18) |  |
| **There is an ongoing FDA required post-approval trial to evaluate safety and effectiveness** | | | |
| Yes | 33 (47) | 15 (20) | <0.001 |
| No – I / the average physician do[es]n’t consider this factor is relevant | 25 (36) | 41 (54) |  |
| No – I / the average physician do[es]n’t have this information | 12 (17) | 20 (26) |  |

^a^You: referring to the physician responding to the survey

Note: Percentages may not add to 100% due to rounding.

**Table H.** **Drug or Medical Device Adverse Event Reporting Behavior, Internists**

|  | **“You”^a^ (N = 99),**  **No. (%)** | **“The average physician in your specialty” (N = 87),**  **No. (%)** | **P-value** |
| --- | --- | --- | --- |
| **If ___ see a patient who has an adverse event that is probably related to a drug or medical device, how likely is it that ___ will report this adverse event?**  **Proportion of physicians reporting “very likely” or “somewhat likely”** | | | |
| To the FDA | 72 (73) | 51 (59) | 0.04 |
| To the manufacturer | 54 (55) | 39 (45) | 0.19 |
| To the health system’s safety reporting system | 89 (90) | 68 (78) | 0.03 |
| **What are the reasons for which ___ might not report that adverse event?** | | | |
| Don’t have time to report | 62 (63) | 77 (89) | <0.001 |
| Didn’t know that I / they should report | 56 (57) | 69 (79) | 0.001 |
| Do not feel it is my / their duty to report | 16 (16) | 33 (38) | <0.001 |
| Don’t know how to report | 67 (68) | 80 (92) | <0.001 |
| Expect patients to report | 13 (13) | 13 (15) | 0.72 |
| **Would ___ answer differently if the patient experienced a medical device-related adverse event compared to a drug-related adverse event?** | | | |
| Yes | 34 (35) | 64 (74) | <0.001 |
| No | 64 (65) | 23 (26) |  |

^a^You: referring to the physician responding to the survey

**Table I.** **Drug or Medical Device Adverse Event Reporting Behavior, Cardiologists**

|  | **“You”^a^ (N = 69),**  **No. (%)** | **“The average physician in your specialty” (N = 77),**  **No. (%)** | **P-value** |
| --- | --- | --- | --- |
| **If ___ see a patient who has an adverse event that is probably related to a drug or medical device, how likely is it that ___ will report this adverse event?**  **Proportion of physicians reporting “very likely” or “somewhat likely”** | | | |
| To the FDA | 46 (67) | 39 (51) | 0.05 |
| To the manufacturer | 52 (75) | 45 (58) | 0.03 |
| To the health system’s safety reporting system | 58 (84) | 61 (79) | 0.45 |
| **What are the reasons for which ___ might not report that adverse event?** | | | |
| Don’t have time to report | 51 (74) | 64 (83) | 0.17 |
| Didn’t know that I / they should report | 33 (48) | 61 (79) | <0.001 |
| Do not feel it is my / their duty to report | 7 (10) | 37 (48) | <0.001 |
| Don’t know how to report | 49 (71) | 71 (92) | <0.001 |
| Expect patients to report | 5 (7) | 4 (5) | 0.61 |
| **Would ___ answer differently if the patient experienced a medical device-related adverse event compared to a drug-related adverse event?** | | | |
| Yes | 32 (46) | 53 (69) | 0.006 |
| No | 37 (54) | 24 (31) |  |

^a^You: referring to the physician responding to the survey

**Table J.** **Drug or Medical Device Adverse Event Reporting Behavior, Oncologists**

|  | **“You”^a^ (N = 71),**  **No. (%)** | **“The average physician in your specialty” (N = 75),**  **No. (%)** | **P-value** |
| --- | --- | --- | --- |
| **If ___ see a patient who has an adverse event that is probably related to a drug or medical device, how likely is it that ___ will report this adverse event?**  **Proportion of physicians reporting “very likely” or “somewhat likely”** | | | |
| To the FDA | 43 (61) | 29 (39) | 0.008 |
| To the manufacturer | 43 (61) | 31 (41) | 0.02 |
| To the health system’s safety reporting system | 55 (77) | 49 (65) | 0.11 |
| **What are the reasons for which ___ might not report that adverse event?** | | | |
| Don’t have time to report | 50 (70) | 71 (95) | <0.001 |
| Didn’t know that I / they should report | 42 (59) | 62 (83) | 0.002 |
| Do not feel it is my / their duty to report | 10 (14) | 44 (59) | <0.001 |
| Don’t know how to report | 44 (62) | 66 (88) | <0.001 |
| Expect patients to report | 1 (1) | 5 (7) | 0.11 |
| **Would ___ answer differently if the patient experienced a medical device-related adverse event compared to a drug-related adverse event?^b^** | | | |
| Yes | 27 (39) | 53 (71) | <0.001 |
| No | 43 (61) | 22 (29) |  |

^a^You: referring to the physician responding to the survey

^b^N = 70 in “You” column
